# The mediating role of inflammation and nutrition metabolism in the methylmalonic acid-stroke association

**DOI:** 10.1016/j.clinsp.2026.101055

**Published:** 2026-07-16

**Authors:** Ningyu Wei, Boyi Tang, Yuan Cheng

**Affiliations:** a Department of Neurosurgery, The Second Affiliated Hospital of Chongqing Medical University, Yuzhong, Chongqing, China; b Department of Radiology, Children's Hospital of Chongqing Medical University, Chongqing, China

**Keywords:** Methylmalonic acid, Stroke, MMA, NHANES, Ischemic stroke

## Abstract

**Background:**

This study explored the association between Methylmalonic Acid (MMA, the primary exposure factor) and stroke, and the potential mediating roles of systemic inflammatory marker (Neutrophil-to-Lymphocyte Ratio [NLR]) and nutritional-metabolic parameters (Triglycerides [TG], Fasting Plasma Glucose [FPG]). Based on biological plausibility and evidence-based rationale, these three indicators were selected a priori and incorporated as core mediating variables in the analytical model.

**Methods:**

We included 9430 participants from NHANES 2011–2014, comprising 712 stroke cases and 8718 non-stroke controls; this cross-sectional study enrolled adults ≥ 20-years. Weighted Multivariable Logistic Regression (WMLR) analyzed MMA-stroke prevalence association; RCS examined their non-linearity. Subgroup/mediation analyses evaluated association heterogeneity and NLR/TG/FPG’s mediating effects. In addition, the mediation effects of other inflammatory, nutritional, metabolic, and oxidative stress-related markers were also evaluated.

**Results:**

WMLR showed a significant positive association between MMA and stroke (continuous MMA: OR [95% CI]: 1.423 [1.166, 1.942]). When MMA was categorized by tertiles (T2: OR = 1.467, 95% CI: 1.067–2.129; T3: OR = 1.644, 95% CI: 1.155–2.578), the association persisted after adjusting for renal function, vitamin B₁₂, and other covariates. RCS indicated a non-linear relationship between MMA and stroke (cut-off point: ln (MMA) = 5). The association was more pronounced in participants aged < 60 years (P for interaction = 0.013). NLR, TG, and FPG exerted partial mediating effects (4.923%, 7.187%, 5.331%; all p < 0.001). By contrast, other inflammatory markers (SIRI, PLR, SII, MLR, albumin, uric acid, LDH) did not exhibit significant mediating effects, with all PM values < 1% and p > 0.05. Given the cross-sectional nature of this study, we cannot determine whether elevated MMA levels preceded stroke or were a consequence of post-stroke pathophysiology.

**Conclusion:**

Serum MMA is positively associated with stroke prevalence, partially mediated by inflammatory response (NLR) and nutritional/metabolic indicators (TG and FPG).

## Highlights

• Elevated MMA levels are associated with increased stroke risk in US adults.
• Restricted cubic spline analysis identified a nonlinear relationship between MMA and stroke, with a cutoff at ln (MMA = 5).
• The MMA-stroke correlation is stronger in younger adults than in older adults (P for interaction = 0.013).
• Combined detection of NLR, TG, and FPG may help identify populations at elevated stroke risk in this cross-sectional exploratory analysis.

## Introduction

According to the latest data from the 2025 Global Burden of Diseases (GBD) report, stroke (including Ischemic Stroke [IS] and Hemorrhagic Stroke [HS]) is the global second leading cause of death,^1^ and the third leading cause of Disability-Adjusted Life Year (DALY) loss. Stroke is mainly composed of IS (65.3%) and intracerebral hemorrhage (28.8%).^2^ The World Stroke Organization (WSO) further pointed out that by 2025, the economic cost related to stroke had exceeded 890 billion US dollars (accounting for 0.66% of global GDP), imposing a significant burden on global public health.^3^ Although traditional risk factors such as hypertension, diabetes, dyslipidemia, and cardiovascular diseases are clearly associated with stroke, they can only explain approximately 50%–60% of stroke cases. A considerable proportion of stroke cases (Cryptogenic Ischemic Stroke [CIS] and early-onset stroke) cannot be explained by these factors.^4^, ^5^, ^6^ Therefore, identifying potential high-risk populations and implementation of targeted prevention strategies are crucial for reducing the overall disease burden of stroke.

Methylmalonic Acid (MMA) is a key intermediate metabolite in the propionic acid metabolism pathway, which is tightly regulated by vitamin B₁₂ (cobalamin). Vitamin B₁₂ deficiency often leads to elevated MMA levels. Elevated MMA levels not only disrupt normal metabolic processes but also inhibit mitochondrial function. This inhibition occurs by impairing mitochondrial respiratory chain function and inducing Reactive Oxygen Species (ROS) production, thereby playing a key role in oxidative stress. Such metabolic disorders can also activate inflammatory pathways and promote thrombosis, collectively contributing to the occurrence of stroke.^7^ Clinical studies have confirmed that abnormally elevated MMA is closely associated with various chronic diseases, such as Chronic Kidney Disease (CKD), atherosclerosis, cardiovascular disease, and diabetes.^8^ Notably, MMA levels correlate strongly with vitamin B₁₂ status and estimated Glomerular Filtration Rate (eGFR): individuals with lower vitamin B₁₂ levels and impaired renal function typically exhibit significantly higher MMA levels. Given that MMA can act on key targets such as blood vessels, myocardium, and metabolic regulatory systems, and its abnormal levels often appear earlier than the clinical symptoms of stroke and related diseases, monitoring baseline MMA levels may have potential utility for assessing stroke risk in healthy or high-risk populations.^9^ Therefore, targeted interventions for elevated MMA (e.g., vitamin B₁₂ supplementation to improve metabolism, application of antioxidant therapy to reduce tissue damage) are expected to provide new directions for the early prevention and treatment of stroke and related diseases.

Currently, epidemiological evidence regarding the association between MMA and stroke prevalence in the general population remains limited. Although some studies have suggested that its mechanism may involve the activation of inflammatory responses and metabolic disturbances, this pathway has not been fully validated in the general population. In particular, among young adults (< 60-years-old), the evidence regarding the association between MMA and stroke remains relatively scarce. This is mainly related to the unique distribution of stroke risk factors in young adults; the high prevalence of risk factors such as sedentary lifestyle and asymptomatic hypertension significantly increases the risk of stroke in this population.^10^ Additionally, approximately 30%–40% of young adult stroke patients have no definite history of exposure to traditional risk factors, and such cases are mostly classified as Cryptogenic Ischemic Stroke (CIS), which makes it difficult for traditional risk assessment models to achieve accurate identification of at-risk individuals and stratified management in this population.^11^, ^12^, ^13^

In summary, this cross-sectional study used data from the National Health and Nutrition Examination Survey (NHANES) to analyze the association between serum MMA and stroke prevalence in American adults. We focused on the young adult subgroup, aiming to explore the utility of MMA as a potential stroke risk marker ‒ particularly in patients with Cryptogenic Ischemic Stroke (CIS) who lack definite traditional risk factors ‒ and to further investigate the mediating roles of inflammatory and nutritional indicators. The study aims to provide evidence-based support for the early risk identification and targeted prevention of stroke.

## Methods

### Study population

**Fig. 1:**
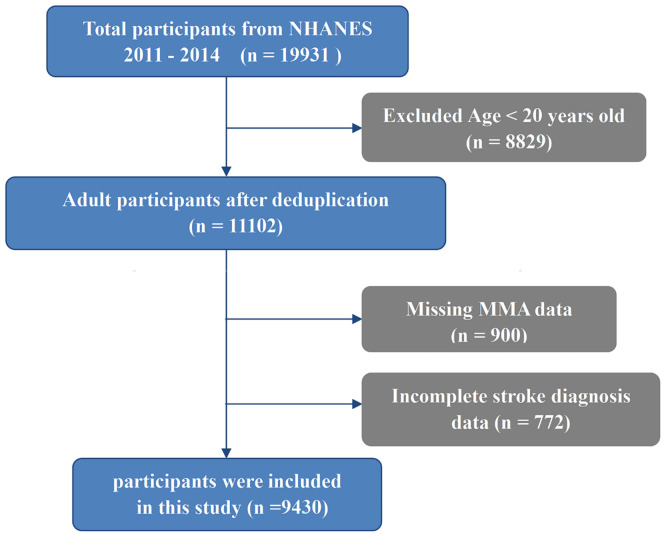
A flowchart for the patient selection process.

This observational cross-sectional study was reported in accordance with the STROBE guidelines. All data were retrieved from the National Health and Nutrition Examination Surveys (NHANES) ‒ a U.S. Centers for Disease Control and Prevention (CDC) ‒ administered program. As reported in prior studies, NHANES is an ongoing national-level stratified multistage probability sampling program designed to assess the health status of non-institutionalized civilian populations across all age groups.^14^ For the current investigation, 11,102 participants aged ≥ 20-years from two 2-year cycles of NHANES (2011–2012 and 2013–2014) were initially included. We excluded individuals with missing valid MMA data (n = 900), incomplete data on stroke diagnosis (n = 772), along with those lacking values for key inflammatory markers (Neutrophil-to-Lymphocyte Ratio [NLR], Systemic Inflammatory Response Index [SIRI], Platelet-to-Lymphocyte Ratio [PLR], Systemic Immune-Inflammation Index [SII], Monocyte-to-Lymphocyte Ratio [MLR]) and metabolic parameters (Triglycerides [TG], Fasting Plasma Glucose [FPG]). Ultimately, 9430 participants were available for the final analysis (Fig. 1).

The study protocol was reviewed and approved by the Institutional Review Board (IRB) of the National Center for Health Statistics (NCHS). All enrolled participants provided written informed consent.

### Research variables

#### Stroke diagnosis criteria

Ascertainment of Stroke Cases: In this study, we employed a literature-validated, multi-source strategy to identify stroke cases utilizing data from the NHANES 2011–2014 cycles. This approach integrated self-reported physician diagnoses and prescription medication profiles (incorporating generic drug names and reported clinical indications). Case ascertainment was based on the following criteria: 1) Self-reported diagnosis: Participants responding “yes” to the Medical Conditions questionnaire item, “Has a doctor or other health professional ever told you that you had a stroke?” (NHANES variable: MCQ160F); and 2) Medication proxies: The reported use of antiplatelet agents (e.g., aspirin, clopidogrel, ticagrelor) or anticoagulants (e.g., warfarin, dabigatran, rivaroxaban) explicitly indicated for the secondary prevention of stroke, derived from the NHANES Prescription Medications questionnaire (RXQ).

To resolve potential discrepancies between these data sources, we applied a hierarchical adjudication rule prioritizing the self-reported physician diagnosis. For individuals with relevant prescription records but no explicit self-reported stroke history, medication indications were inferred based on established clinical guidelines for stroke prevention, in combination with self-reported cardiovascular comorbidities (coronary artery disease, atrial fibrillation) available in the NHANES dataset. Cases were included only if the medication use was consistent with secondary stroke prevention, thereby minimizing misclassification bias.

#### MMA measurement

Venous blood samples were obtained via venipuncture at Mobile Examination Centers (MEC) in line with standardized NHANES procedures. MMA was measured in plasma and/or serum (plasma as the preferred matrix), with serum and plasma concentrations sharing a common reference range and comparable Coefficients of Variation (CV).^15^

Serum MMA levels were quantified using Isotope-Dilution Liquid Chromatography-tandem Mass Spectrometry (ID-LC-MS/MS) following derivatization with butanol, as detailed in the NHANES Laboratory Procedures Manual.^16^ Briefly, 275 µL of specimens were spiked with an internal standard solution containing isotope-labeled Methyl-d3-Malonic Acid (d3MMA) and subjected to extraction. They were subsequently derivatized with cyclohexanol to form dicyclohexyl ester. After completion of derivatization and separation steps, the effluent was monitored using a mass-selective detector via selected ion monitoring. MMA concentrations were quantified based on the peak area ratio of endogenous MMA to isotope-labeled d3MMA. The assay demonstrated a favorable linear relationship over the concentration range of 50–2000 nmoL/L, with a total CV ranging from 4% to 10% and a mean recovery rate of 96.0%±1.9%. Samples with MMA concentrations exceeding 400 nmoL/L underwent duplicate analysis, consistent with NHANES laboratory protocols.

#### Mediating variable

In this study, we investigated the potential mechanisms underlying the pathogenesis of stroke across three core biological pathways (inflammation, nutritional metabolism, and oxidative stress) via mediation effect analysis. To avoid post-hoc bias, all candidate mediators were selected a priori based on biological plausibility and existing literature. Specifically, we predefined NLR, TG, and FPG as primary mediators, as they closely reflect systemic inflammation, lipid metabolism disturbance, and glucose dysregulation ‒ factors potentially linked to stroke-related biological pathways.^17^, ^18^, ^19^ Concurrently, a secondary panel of relevant biomarkers within these pathways was pre-specified for mediation analysis, including inflammatory/immune markers (SIRI, PLR, SII, MLR), a nutritional/metabolic marker (albumin), and oxidative stress markers (uric acid, LDH). To rigorously address the concern of multiple comparisons, the Benjamini-Hochberg False Discovery Rate (FDR) correction was applied across all tested mediators. The finding that only NLR, TG, and FPG remained significant after FDR correction suggests a plausible biological specificity of these markers. By contrast, the non-significant results for other secondary markers may be attributable to relatively modest effect sizes and potential type II error.

#### Definition of covariates

General information on age, sex, race/ethnicity, family income, smoking status, physical activity, and alcohol consumption was collected using standardized questionnaires during interviews. Demographic information included sex (female and male), age, race (Mexican American, other Hispanic, non-Hispanic Black, non-Hispanic White, and other races), education level (Below high school, High school and Above), marital status (Never married, Married, Formerly married), and household Poverty-to-Income Ratio (PIR) classified as low income (PIR ≤ 1.30), moderate income (PIR 1.31–3.50), and high income (PIR ≥ 3.50). Physical examination included blood pressure and Body Mass Index (BMI). BMI categories followed the World Health Organization (WHO) guidelines: normal or underweight (< 24.9 kg/m^2^), overweight (25.0–29.9 kg/m^2^), and obese (≥ 30.0 kg/m^2^). Laboratory test indicators included fasting glucose, glycated Hemoglobin (HbA1c), red blood cell count, hemoglobin, platelet count, total cholesterol, as well as standard biochemical indicators such as total protein, blood glucose, triglycerides, sodium, potassium, chloride, and total calcium. Information on behaviors and comorbidities was collected via questionnaires. Smoking status was classified as current smoker (having smoked ≥ 100 cigarettes in a lifetime and currently smoking), former smoker (having smoked ≥ 100 cigarettes but having quit), and never smoker (having smoked < 100 cigarettes). Alcohol consumption status was classified as drinker (having a history of drinking or drinking within the past year) and non-drinker. Physical activity was assessed using the Global Physical Activity Questionnaire (NHANES variables: PAQ605–PAQ680). Following the GPAQ analysis guide, total weekly physical activity (MET-minutes/week) was calculated by summing MET-minutes from work-related activities, transportation, and recreational activities. Participants were then classified into inactive (< 600 MET-minutes/week) and active (≥ 600 MET-minutes/week). Comorbidities included diabetes, hypertension, and hyperlipidemia. Diabetes was defined as meeting any of the following criteria: self-reported doctor diagnosis, current use of insulin or oral hypoglycemic agents, fasting glucose ≥ 126 mg/dL, or HbA1c ≥ 6.5%. Hypertension was defined as self-reported doctor diagnosis, currently achieving blood pressure control goals, or measured systolic blood pressure ≥ 140 mmHg or diastolic blood pressure ≥ 90 mmHg. Hyperlipidemia was determined based on self-reported doctor diagnosis or current use of cholesterol-lowering drugs.

### Statistical analysis

This study was conducted in accordance with the complex sampling design of the NHANES database. We incorporated official sampling weights, stratification, and clustering variables into all statistical analyses to ensure that the present results were representative of the target general population. Before statistical analysis, serum MMA concentrations were subjected to natural logarithm transformation (expressed as ln [MMA]) to reduce skewness. After normality testing of continuous variables, those conforming to a normal distribution were expressed as mean ± standard deviation (Mean ± SD); inter-group comparisons for these variables were performed using analysis of variance (ANOVA). Continuous variables with a non-normal distribution were expressed as median (interquartile range) (Median [IQR]). Inter-group comparisons for these variables were conducted using the Kruskal-Wallis test. Categorical variables were expressed as frequency (percentage) [n (%)], and inter-group comparisons were performed using the Chi-Square test (χ² test) or Fisher’s exact test. Multicollinearity between variables was assessed using the Variance Inflation Factor (VIF) in all analyses. To ensure data quality, variables with over 20% missing data were excluded. The remaining missing data were treated utilizing the Multiple Imputation by Chained Equations (MICE) method with random forests. This approach effectively preserves the original distribution of the data.^20^

Weighted Multivariable Logistic Regression (WMLR) models were used to analyze the association between MMA and stroke prevalence. Adjusted Odds Ratios (Adjusted OR) and their 95% Confidence Intervals (95% CI) were calculated, and confounding factors were adjusted in layers as follows: Model 1: No adjustment; Model 2: Further adjustment for demographic characteristics (sex, age, education level, race, marital status, and household income); Model 3: On the basis of Model 2, additional adjustment for clinically relevant variables (BMI, smoking, alcohol consumption, physical activity, diabetes, hypertension, and hyperlipidemia); Model 4: On the basis of Model 3, eGFR was further incorporated. Given that MMA is mainly eliminated via the kidneys, renal function status is a key factor affecting its levels, and renal function decline itself is an independent risk factor for stroke, the inclusion of eGFR in this model is intended to exclude the potential confounding effect of renal impairment. eGFR was calculated using the Chronic Kidney Disease Epidemiology Collaboration (CKD-EPI) formula,^21^ which is the recommended standard for adult renal function assessment in the guidelines of the Kidney Disease: Improving Global Outcomes (KDIGO).^22^ Sequential adjustment was performed in Models 4 and 5 to assess incremental confounding by eGFR and serum vitamin B₁₂, respectively. This stepwise approach allowed us to quantify the independent contribution of each factor to the MMA-stroke association. It also helped evaluate whether the association remained robust after accounting for these key biological correlates of MMA. Model 5: On the basis of Model 4, serum vitamin B₁₂ was further incorporated to control for the potential interference of vitamin B₁₂ deficiency on MMA metabolism and stroke risk. The continuous variable ln (MMA) and the categorical variable of MMA grouped by tertiles (T1–T3, with T1 as the reference group) were included in the models separately.

RCS was used to fit the non-linear relationship between MMA and stroke, while subgroup analyses were conducted to test the robustness of the results and evaluate potential variable interactions. All subgroup analyses (including those stratified by age, race, marital status, creatinine level, lifestyle factors, and comorbidities) were exploratory, conducted to assess the generalizability of primary findings, and should be interpreted with caution due to the risk of chance findings from multiple testing. Mediation analysis was performed to verify the potential mediating roles of both primary (NLR, TG, FPG) and secondary biomarkers (SIRI, PLR, SII, MLR, albumin, uric acid, and LDH). Based on 1000 bootstrap resamplings, the Total Effect (TE), Average Direct Effect (ADE), Average Mediation Effect (AME), and Proportion Mediated (PM) were calculated to evaluate the pathway roles of inflammation, nutritional status, and oxidative stress in the association between MMA and stroke. To rigorously control for false positives arising from multiple comparisons across all tested mediators, p-values were adjusted using the Benjamini-Hochberg False Discovery Rate (FDR) method. Mediation effects with an FDR-adjusted p-value (q-value) < 0.05 were considered statistically significant.

All statistical analyses were performed in R 4.5.1. Two-sided tests were applied, and p < 0.05 was considered statistically significant.

## Results

### Baseline characteristics of the patients

We included 9430 participants from NHANES 2011‒2014, comprising 712 (weighted prevalence 8.1%) stroke cases and 8718 non-stroke controls. The median serum MMA concentration was 147.00 nmol/L (IQR: 112–189 nmol/L). The median age of the participants was 49-years, with 5012 males (51%), and non-Hispanic Whites being the majority (69%, n = 4434). Participants were divided into three groups according to MMA tertiles: T1 group (< 118 nmol/L), T2 group (118–168 nmol/L), and T3 group (> 168 nmol/L). Detailed baseline characteristics of each group are presented in Table 1. Compared with the T1 group (low MMA), participants in the T3 group (high MMA) had significantly higher levels of albumin (g/dL), alkaline phosphatase (U/L), blood urea nitrogen (mg/dL), creatinine (mg/dL), uric acid (mg/dL), and triglycerides (mg/dL) (all p < 0.05). Additionally, the T3 group had a higher proportion of smokers, drinkers, individuals with inactive physical activity, and patients with diabetes, hypertension, and hyperlipidemia (all p < 0.05).

**Table 1:** Baseline characteristics of Stroke patients grouped by variates.

**Characteristic**	**Overall**	**T1**	**T2**	**T3**	**p-value^b^**
	**n=9,430^a^**	**n=9,430^a^**	**n=9,430^a^**	**n=9,430^a^**	
**Gender**					0.022
Male	5012 (51%)	1982 (56%)	1382 (48%)	1927 (53%)	
Female	4289 (47%)	1738 (46%)	1937(52%)	1488 (47%)	
**Age**	49 (32,68)	44 (35,55)	47 (36,62)	57 (43,69)	<0.001
**Race**					<0.001
Mexican American	1395 (8.8%)	578 (16%)	363 (6.7%)	29 8(4.9%)	
Other Hispanic	983 (6.5%)	394 (7.9%)	374 (6.4%)	296 (4.8%)	
Non-Hispanic White	4434 (69%)	885 49%)	1723 (73%)	1934 (78%)	
Non-Hispanic Black	2537 (12%)	985 (18%)	786 (9.7%)	582 (6.7%)	
Other Race ‒ Including Multi-Racial	1575 (7.9%)	671 (12%)	513 (7.7%)	495 (6.5%)	
**Education level**					0.213
Below high school	2634 (18%)	634 (15%)	696 (15%)	884 (18%)	
High school and above	7834 (85%)	2432 (83%)	2741 (86%)	2496 (85%)	
**Marital status**					<0.001
Formerly married	2329 (19%)	581 (14%)	701 (19%)	1428 (26%)	
Married	5527 (62%)	1912 (63%)	2549 (67%)	1914 (63%)	
Never married	1912 (19%)	717 (23%)	681 (18%)	482 (14%)	
**INDFMPIR**					<0.001
> 3.50	3012 (41%)	1023 (40%)	1126 (44%)	961 (40%)	
≤ 1.30	3424 (25%)	1087 (27%)	1102 (21%)	1328 (25%)	
1.31‒3.50	3287 (34%)	1134 (32%)	1265 (34%)	1211 (35%)	
**BMI**					0.256
Obese	3265 (35%)	1365 (39%)	1387 (35%)	1208 (36%)	
Overweight	3301 (34%)	1156 (33%)	1148 (35%)	1129 (35%)	
Under or normal weight	3023 (29%)	992 (28%)	1038 (30%)	1016 (29%)	
**Smoke**					<0.001
Former	2312 (25%)	671 (22%)	912 (26%)	926 (27%)	
Never	5591 (56%)	2029 (60%)	1871(55%)	1761 (53%)	
Now	1923 (19%)	691 (18%)	687 (20%)	693 (20%)	
**Drink**	8778 (89%)	2831 (89%)	2912 (90%)	2576 (88%)	0.048
**Diabetes**	1698 (13%)	486 (11%)	560 (12%)	764 (15%)	<0.001
**Hypertension**	3914 (34%)	951 (27%)	1384 (32%)	1935 (44%)	<0.001
**Hyperlipidemia**	3641 (37%)	1123 (31%)	1233 (35%)	1788 (44%)	<0.001
**Physical activity**					<0.001
Active	5950 (63%)	2086 (66%)	2064 (65%)	1800 (58%)	
Inactive	4154 (36%)	1186 (34%)	1316 (37%)	1724 (43%)	
**Stroke**	712 (8.1%)	434 (6.6%)	458 (8.6%)	347 (11.0%)	<0.001
**Blood Urea Nitrogen (mg/dL)**	12 (10,16)	11 (9,14)	13 (10,15)	14 (11.18)	<0.001
**Alanine aminotransferase ALT (U/L)**	21 (15,28)	21 (16,29)	21 (15,29)	20 (16,26)	0.0123
**Bicarbonate (mmol/L)**	25 (24,27)	25 (23,26)	25 (24,27)	25 (23,27)	<0.001
**Blood sugar, serum (mg/dL)**	93 (85,104)	92 (85,101)	93 (85,105)	94 (86,107)	<0.001
**Iron, Remastered (μg/dL)**	82 (62,106)	81 (60,107)	83 (65,108)	80 (61,103)	0.032
**Estimated GFR (eGFR, mL/min/1.73m²)^c^**	90.1 ± 12.3	94.6 ± 11.2	89.8 ± 10.5	85.2 ± 13.1	
**Creatine phosphokinase (CPK) (IU/L)**	102 (71,157)	103 (72,163)	104 (74,160)	99 (68,148)	<0.001
**Triglycerides (mg/dL)**	121 (80,186)	111 (74,173)	123 (78,189)	128 (86,200)	<0.001
**Methylmalonic acid (nmol/L)**	147 (112,189)	99 (84,109)	143 (129,158)	214 (185,274)	<0.001
**Osmotic pressure (mmol/kg)**	278 (276,281)	277 (275,280)	278 (275,282)	279 (276,283)	<0.001
**Lactate dehydrogenase (U/L)**	123 (109,140)	121 (107,136)	122 (109,138)	125 (111,143)	<0.001
**Platelet count (1000 cells/μL)**	229 (196,270)	234 (202,273)	229 (197,270)	225 (190,268)	<0.001
**Chloride (mmol/L)**	104 (102,106)	104 (103,106)	104(102,106)	104 (102,106)	<0.001
**Cholesterol (mg/dL)**	190 (163,218)	189 (164,217)	190(164,217)	191 (163,219)	0.534
**Sodium (mmol/L)**	138 (138,141)	139 (138,140)	139 (138,141)	140 (138,141)	0.015
**Glycated hemoglobin (%)**	5.40 (5.20, 5.79)	5.40 (5.20, 5.70)	5.40 (5.20, 5.76)	5.50 (5.20, 5.90)	<0.001
**Albumin(g/dL)**	4.25 (4.11, 4.49)	4.28 (4.12, 4.50)	4.30 (4.15, 4.50)	4.30 (4.04, 4.51)	<0.001
**Creatinine (mg/dL)**	0.87 (0.71, 1.01)	0.79 (0.67, 0.93)	0.86 (0.73, 0.98)	0.91 (0.76, 1.09)	<0.001
**Total protein (g/dL)**	7.05 (6.70, 7.40)	7.10 (6.80, 7.40)	7.04 (6.80, 7.31)	7.00 (6.70, 7.30)	<0.001
**Total bilirubin (mg/dL)**	0.59 (0.50, 0.80)	0.59 (0.50, 0.80)	0.60 (0.50, 0.80)	0.60 (0.50, 0.80)	0.023
**Total calcium (mg/dL)**	9.40 (9.20, 9.60)	9.4 0(9.20, 9.60)	9.40 (9.20, 9.60)	9.40 (9.20, 9.60)	0.011
**Phosphorus (mg/dL)**	3.80 (3.40, 4.15)	3.70 (3.40, 4.10)	3.72 (3.40, 4.14)	3.80 (3.40, 4.20)	<0.001
**Uric acid (mg/dL)**	5.28 (4.40, 6.29)	5.10 (4.20, 6.00)	5.30 (4.40, 6.31)	5.52 (4.60, 6.50)	<0.001
**Potassium (mmol/L)**	4.00 (3.80, 4.20)	3.90 (3.70, 4.10)	4.00 (3.80, 4.20)	4.00 (3.80, 4.20)	<0.001
**Globulin (g/dL)**	2.70 (2.50, 3.00)	2.80 (2.50, 3.10)	2.70 (2.50, 3.05)	2.70 (2.50, 3.00)	<0.001
**White blood cell count (1000 cells/μL)**	6.90 (5.70, 8.40)	6.80 (5.60, 8.30)	6.80 (5.60, 8.35)	7.00 (5.90, 8.40)	<0.001
**Monocyte count (1000 cells/μL)**	0.50 (0.40, 0.70)	0.50 (0.40, 0.64)	0.50 (0.40, 0.64)	0.50 (0.40, 0.70)	<0.001
**Segmented neutrophil count (1000 cells/μL)**	4.00 (3.10, 5.20)	3.90 (3.00, 5.15)	4.00 (3.10, 5.25)	4.10 (3.30, 5.30)	<0.001
**Lymphocyte count (1000 cells/μL)**	2.00 (1.60, 2.50)	2.00 (1.70, 2.50)	2.00 (1.60, 2.50)	2.00 (1.50, 2.40)	0.003
**Red blood cell count (millions of cells/μL)**	0.20 (0.10, 0.24)	0.20 (0.10, 0.20)	0.20 (0.10, 0.24)	0.20 (0.10, 0.30)	0.026
**Hemoglobin (g/dL)**	14.10 (13.20, 15.10)	14.00 (13.10, 15.10)	14.30 (13.40, 15.20)	14.15 (13.10, 15.10)	<0.001

All group comparisons were performed using survey-weighted statistical methods to account for the complex sampling design of NHANES.

MMA, Methylmalonic Acid; PIR, Poverty Income Ratio; BMI, Body Mass Index;.

^a^Median (Q1, Q3); n (unweighted) (%).

^b^Design-based Kruskal-Wallis test; Pearson’s X^2: Rao & Scott adjustment.

^c^eGFR presented as mean ± SD due to normal distribution; all other continuous variables shown as median (IQR).

### Association between MMA and stroke prevalence

In the Results section, we extended this analysis by employing WMLR models to examine the association between serum MMA and stroke prevalence. Confounding factors were adjusted gradually from Model 1 to Model 5. As shown in Table 2, continuous MMA was significantly positively associated with stroke prevalence across all models: Model 1: (OR [95% CI]: 1.643 [1.433, 2.189], p < 0.001); Model 2: (OR [95% CI]: 1.323 [1.256, 1.956], p = 0.008); Model 3: (OR [95% CI]: 1.423 [1.166, 1.942], p = 0.031); Model 4: (OR [95% CI]: 1.357 [1.102, 1.871], p = 0.028); Model 5: (OR [95% CI]: 1.321 [1.085, 1.814], p = 0.032). Similarly, in the tertile-based analysis (with T1 as the reference group), compared with the low MMA level (T1), the stroke prevalence in the moderate (T2) and high (T3) MMA level groups was significantly higher in all five models. Specifically, in Model 1: T2 (OR [95% CI]: 1.477 [1.157, 2.187], p = 0.018); T3 (OR [95% CI]: 2.497 [1.745, 3.478], p < 0.001). In Model 3: (T2: OR [95% CI]: 1.467 [1.067, 2.129], p = 0.041; T3: OR [95% CI]: 1.644 [1.155, 2.578], p = 0.039), Model 4: (T2: OR [95% CI]: 1.392 [1.011, 1.918], p = 0.043; T3: OR [95% CI]: 1.562 [1.094, 2.427], p = 0.036), Model 5: (T2: OR [95% CI]: 1.365 [1.002, 1.883], p = 0.045; T3: OR [95% CI]: 1.528 [1.076, 2.319], p = 0.038).

**Table 2:** Link between MMA and the prevalence of Stroke analyzed utilizing weighted logistic regression.

**Characteristic**	**Model 1**	**Model 2**	**Model 3**	**Model 4**	**Model 5**
**In (MMA)**					
OR (95% CI)	1.643 (1.433, 2.189)	1.323 (1.256, 1.956)	1.423 (1.166, 1.942)	1.357 (1.102, 1.871)	1.321 (1.085, 1.814)
p-value	<0.001	0.008	0.031	0.028	0.032
**MMA grouped (P for trend)**	<0.001	0.013	0.043	0.048	0.049
T1 (Reference)	‒	‒	‒	‒	<0.001
T2	1.477 (1.157, 2.187), p = 0.018	1.422 1.067, 2.167), p = 0.047	1.467 (1.067, 2.129), p = 0.041	1.392 (1.011, 1.918), p = 0.043	1.365 (1.002, 1.883), p = 0.045
T3	2.497 (1.745, 3.478), p < 0.001	1.755 (1.277, 2.589), p = 0.034	1.644 (1.155, 2.578), p = 0.039	1.562 (1.094, 2.427), p = 0.036	1.528 (1.076, 2.319), p = 0.038

All odds ratios (ORs) and 95% Confidence Intervals (95% CIs) were calculated using survey-weighted logistic regression to account for the complex sampling design of NHANES.

Model 1: Unadjusted. Model 2: Adjusted for age, gender and race. Model 3: Adjusted for age, gender, race, BMI, smoking, drinking, physical activity, diabetes, hypertension and hyperlipidemia. Model 4: Adjusted for all covariates in Model 3 and estimated Glomerular Filtration Rate (eGFR). Model 5: Adjusted for all covariates in Model 4 and serum vitamin B₁₂.

**Fig. 2:**
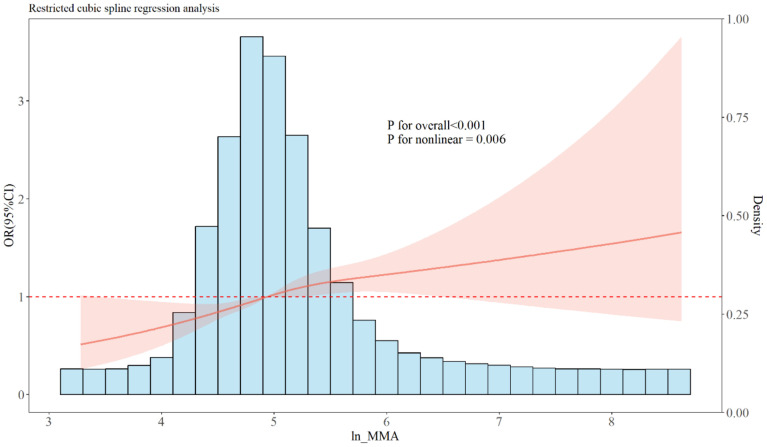
Restricted cubic spline regression analysis of MMA and Stroke with prevalence rate.

RCS curves were used to evaluate the non-linear relationship between MMA and stroke prevalence. The results (Fig. 2) showed a significant non-linear association between them (P for non-linearity < 0.001). When ln (MMA) > 5 (threshold), stroke prevalence increased significantly with the elevation of MMA.

### Mediation analysis

**Fig. 3:**
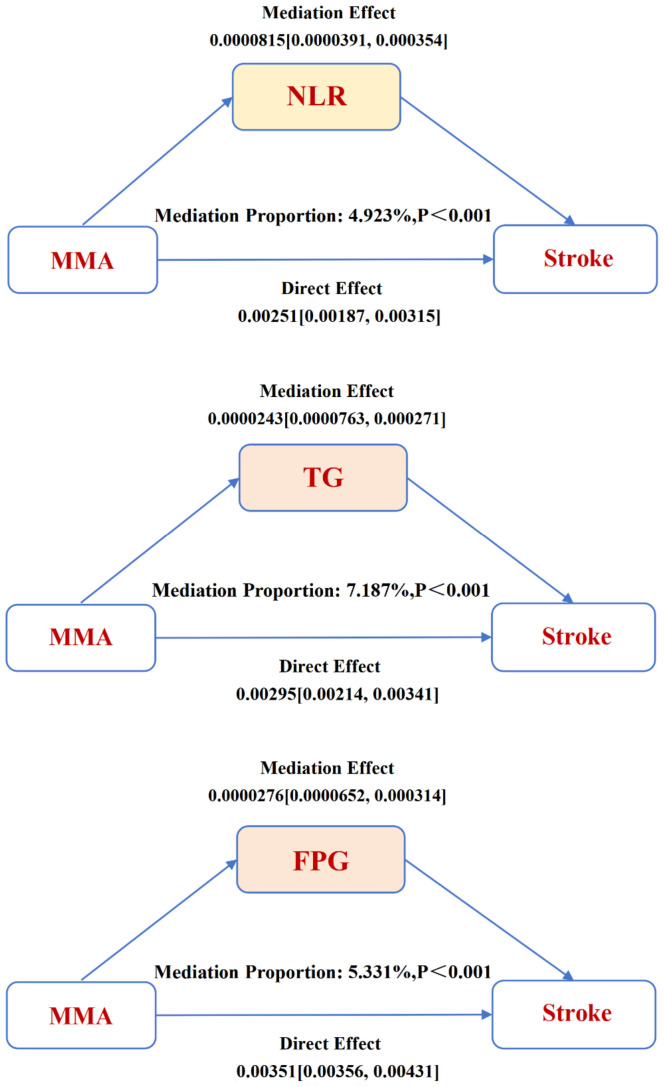
Mediation analysis diagrams illustrating the indirect effects of Methylmalonic Acid (MMA) on stroke through the Neutrophil-to-Lymphocyte Ratio (NLR), Triglycerides (TG), and Fasting Plasma Glucose (FPG).

Mediation analysis was conducted to evaluate the mediating roles of inflammatory (NLR, SIRI, PLR, SII, MLR) and nutritional/metabolic (TG, FPG) indicators in the MMA-stroke association. The results showed that NLR, TG, and FPG had significant partial mediating effects in the association (all p < 0.001), with their mediating effect proportions being 4.923%, 7.187%, and 5.331%, respectively (Fig. 3). However, other inflammatory and metabolic markers (including SIRI, PLR, SII, MLR, albumin, uric acid, and LDH) showed no significant mediating effects (all PM < 1%, p > 0.05).

### Subgroup analysis

**Fig. 4:**
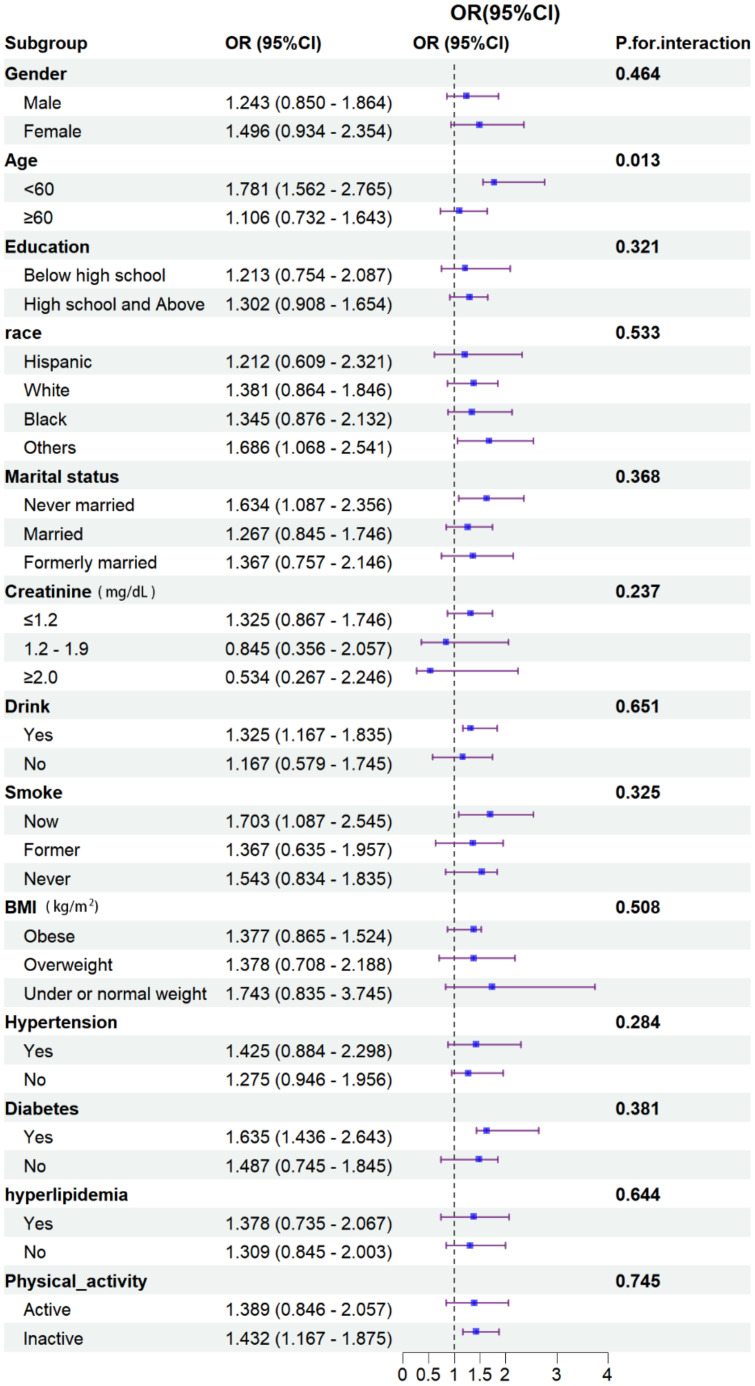
Forest plots of odds ratio for the primary endpoint in different subgroups.

Stratified analysis was conducted to explore the association between MMA and stroke prevalence in different subgroups and to test interactions (Fig. 4). Of these, the age subgroup analysis was pre-specified, while all other subgroup analyses were exploratory in nature. The results showed that MMA was significantly positively associated with stroke prevalence in multiple subgroups, including those stratified by sex, age, education level, race, marital status, creatinine level (mg/dL), alcohol consumption, smoking status, BMI (kg/m^2^), diabetes, hypertension, hyperlipidemia, and physical activity (OR > 1 in all subgroups). Further analysis indicated that this association was particularly prominent in participants of other races, unmarried individuals, current smokers, drinkers, diabetic patients, and those with inactive physical activity. A significant interaction was observed with age (P for interaction = 0.013). The positive association between MMA and stroke was significant in participants aged < 60-years (OR = 1.781, 95% CI: 1.562–2.765) but was not statistically significant in those aged ≥ 60-years (OR = 1.106, 95% CI: 0.732–1.643).

These findings should be interpreted in the context of the study's cross-sectional design, which precludes definitive conclusions about causality or the temporal relationship between MMA and stroke.

## Discussion

Based on the NHANES database, this study explored the association between serum MMA and stroke prevalence, and analyzed the mediating roles of systemic inflammation and nutritional metabolism in this association. The results showed that elevated MMA was positively associated with increased stroke prevalence. RCS results indicated a non-linear relationship between them (cut-off point: ln (MMA) = 5), with stroke prevalence increasing as MMA levels rose. Subgroup analysis revealed a statistically significant interaction between age and MMA, with the association being more pronounced in participants aged < 60-years. Mediation analysis further confirmed that NLR, TG, and FPG partially mediated the association between MMA and stroke prevalence. These exploratory findings highlight potential pathways that warrant further investigation in prospective studies and may inform future research on risk stratification and preventive strategies.

Previous studies have shown that elevated MMA is positively associated with an increased risk of various chronic diseases. Its potential pathogenic mechanism mainly involves the activation of inflammatory response and oxidative stress pathways, which in turn induce mitochondrial dysfunction and accelerate the pathological progression of diseases.^23^, ^24^, ^25^ Consistent with these findings, the present study confirmed a positive association between elevated MMA and stroke prevalence, suggesting that MMA may be linked to stroke pathogenesis via multiple pathways (e.g., inflammation, oxidative stress, vascular injury). Most notably, this analysis reveals a robust and independent association between elevated serum MMA levels and increased stroke prevalence. This association persists even after rigorous adjustment for renal function (eGFR) and vitamin B₁₂ status. This finding is particularly important because prior studies have demonstrated that vitamin B₁₂ and eGFR can only explain a limited proportion (16%–20%) of the variation in MMA concentrations.^26^ Notably, this variance decomposition analysis was not performed in the current dataset; these findings are derived from external epidemiological and clinical studies, which indicates that the association between MMA and stroke risk has independent pathophysiological significance independent of traditional renal function and vitamin B₁₂ metabolic pathways.

All subgroup analyses were exploratory but guided by biological plausibility to preclude unstructured data dredging. While the association was robust across most subgroups and unaffected by comorbidities or lifestyle factors, age emerged as a significant modifier (p = 0.013). This suggests that the relationship between MMA and stroke prevalence is age-dependent, consistent with prior evidence. Furthermore, we observed potentially stronger associations in certain high-risk subgroups, such as smokers, individuals with diabetes, and physically inactive participants. Biologically, this may be explained by a compounded effect of systemic inflammation and endothelial dysfunction prevalent in these populations. Nevertheless, these exploratory subgroup findings should be interpreted cautiously due to the potential risk of chance results from multiple testing.^27^ This finding is consistent with multiple epidemiological studies. A study in Oxfordshire, UK (2002–2018) reported a 67% increase in stroke incidence in individuals aged < 55-years, while a 15% decrease in prevalence was observed in those aged ≥55-years.^28^, ^29^, ^30^ A large-sample study in Fukuoka, Japan (n = 15,860) further pointed out that although the overall incidence of hypertension, diabetes, and dyslipidemia in young adults (< 60-years) is lower than that in the elderly, the prevalence of diabetes and dyslipidemia in individuals aged > 40-years is close to that in the elderly. Moreover, lifestyle risk factors such as smoking, alcohol consumption, and obesity are more common in young populations.^31^, ^32^, ^33^ Collectively, these results suggest that in relatively young populations, due to the early stage of cerebrovascular pathological changes and less accumulation of traditional risk factors, the damaging effects of metabolic disorders (e.g., abnormal TG and FPG) and inflammatory activation (e.g., elevated NLR) reflected by MMA on vascular endothelium may be more prominent.^34^ Therefore, these findings highlight MMA and its associated indicators as promising targets for future risk stratification research.

Mediation analysis suggested that NLR, TG, and FPG may play partial mediating roles in the observed association between MMA and stroke prevalence. In terms of the inflammatory pathway, elevated NLR reflects an inflammatory state characterized by a relative increase in neutrophils and a relative decrease in lymphocytes. This state can induce vascular endothelial injury and promote the formation of atherosclerotic plaques. Multiple studies have further confirmed that NLR levels in stroke patients are significantly higher than those in healthy controls, and NLR levels in patients with moderate-to-severe stroke are also significantly higher than those in patients with mild stroke.^35^ In terms of the metabolic pathway, the mediating role of TG suggests that MMA may disrupt lipid metabolism. This disruption can increase TG levels, thereby promoting the accumulation of triglyceride-rich lipoproteins in the blood. Studies have shown that high TG levels are positively associated with the risk of IS, and the risk of IS in individuals with TG > 5 mmol/L is approximately 3-times that in individuals with TG < 1 mmol/L.^36^ The mediating role of FPG suggests that MMA may interfere with the insulin signaling pathway. This interference can induce insulin resistance and elevated blood glucose, which in turn increases blood viscosity and promotes thrombosis. Studies have shown that in individuals with FPG ≥6.1 mmol/L, the risk of overall stroke events increases by 6% for every approximately 0.56 mmol/L increase in blood glucose.^37^ To ensure the robustness of these findings, all predefined primary and secondary biomarkers were subjected to rigorous multiple testing correction. Following FDR correction, only NLR, TG, and FPG demonstrated significant mediation effects, highlighting their potential role in the inflammatory and metabolic pathways linking MMA to stroke risk. Notably, the non-significant mediation effects observed for secondary markers (such as SIRI, albumin, etc.) do not strictly rule out their biological involvement; rather, this null result may be attributable to smaller effect sizes in the studied cohort, biological variance, and potential type II error.

Furthermore, it is important to note that while NLR, TG, and FPG showed statistically significant mediation, their effect sizes were modest (explaining approximately 4.9%–7.2% of the effect). The fact that > 90% of the total effect remains unexplained indicates that the association between MMA and stroke is largely driven by other direct or indirect mechanisms not captured in this model. This highlights the complex pathophysiology of stroke and warrants further investigation into alternative pathways, such as direct vascular endothelial toxicity, impaired renal clearance, and thrombosis.

The present study is subject to several limitations. First, the cross-sectional design precludes inferring a causal relationship between MMA and stroke, and the MMA level based on a single measurement cannot reflect its long-term changes, with the results potentially affected by short-term fluctuations. Second, reliance on self-reported medication history may introduce recall bias, and excluding participants with incomplete data could limit the generalizability of these findings. Although we adjusted for key covariates including age, comorbidities, renal function, and vitamin B₁₂ levels, residual confounding remains inevitable. Potential unmeasured confounders, such as a history of neurological diseases and homocysteine levels, may also influence the observed associations. Notably, homocysteine data were unavailable in the NHANES 2011–2014 dataset, precluding the analysis of its interaction with MMA. Future prospective studies incorporating comprehensive metabolic profiles are warranted to validate these findings. Third, the causal interpretation of mediation analysis is limited by the inherent constraints of cross-sectional data, which relies on strong and unverifiable assumptions. Specifically, cross-sectional mediation assumes the correct temporal ordering of variables (i.e., the exposure strictly precedes the mediator, which in turn precedes the outcome), an assumption that cannot be definitively established in the present study design. Furthermore, it assumes the absolute absence of unmeasured confounding across the exposure-mediator, mediator-outcome, and exposure-outcome relationships. It must be emphasized that even with well-measured and comprehensively adjusted covariates, residual confounding cannot be completely ruled out in such an observational setting. Therefore, the identified mediating pathways should be considered indicative and hypothesis-generating rather than definitively causal.

Future studies should conduct multi-center, large-sample prospective cohort studies to verify the associative relevance of MMA for different stroke subtypes (e.g., ischemic stroke, hemorrhagic stroke) through long-term follow-up, and clarify the target population and clinical threshold of MMA as a stroke predictive indicator. Molecular biology techniques (e.g., mitochondrial function detection, metabolomics analysis) should be used to further explore the specific molecular mechanisms by which MMA affects stroke occurrence through mitochondrial dysfunction and nutritional metabolism disorders. Additionally, based on the MMA cut-off point identified in this study, the preventive effect of reducing MMA (e.g., vitamin B₁₂ supplementation) on stroke should be verified.

## Conclusion

In summary, the present study demonstrates a significant positive association between elevated serum MMA concentrations and increased stroke prevalence, an association that is more pronounced in individuals aged < 60-years. The findings further identify NLR, TG, and FPG as potential mediators, indicating that the observed association may be partially mediated via inflammatory and metabolic pathways.

## Abbreviations

ACME, Average Causal Mediation Effect; ADE, Average Direct Effect; BMI, Body Mass Index; CDC, Centers for Disease Control and Prevention; CI, Confidence Interval; CIS, Cryptogenic Ischemic Stroke; DALY, Disability-Adjusted Life Year; GBD, Global Burden of Diseases; HS, Hemorrhagic Stroke; ID-LC-MS/MS, Isotope Dilution Liquid Chromatography-Tandem Mass Spectrometry; IS, Ischemic Stroke; LOD, Limit of Detection; MLR, Monocyte-to-Lymphocyte Ratio; MMA, Methylmalonic Acid; NHANES, National Health and Nutrition Examination Survey; NCHS, National Center for Health Statistics; NLR, Neutrophil-to-Lymphocyte Ratio; OR, Odds Ratio; PLR, Platelet-to-Lymphocyte Ratio; PM, Proportion Mediated; RCS, Restricted Cubic Spline; SIRI, Systemic Inflammation Response Index; TE, Total Effect; WMLR, Weighted multivariable logistic regression; WSO, World Stroke Organization.

## Authors’ contributions

Ningyu Wei: Conceptualization; Formal analysis; Investigation; Resources; Writing – original draft; Writing – review & editing. Boyi Tang: Conceptualization; Methodology; Writing – review & editing. Yuan Cheng: Conceptualization; Supervision; Writing – review & editing. All authors have read and approved the final manuscript.

## Ethical statement

Not applicable.

## Funding

This research did not receive any specific grant from funding agencies in the public, commercial, or not-for-profit sectors.

## Data availability

The corresponding author can be contacted to receive the datasets generated and utilized in this work upon reasonable request and with NHANES’s permission.

## Conflicts of interest

The authors declare no conflicts of interest.
